# Exploring the performance of automatic speaker recognition using twin speech and deep learning-based artificial neural networks

**DOI:** 10.3389/frai.2024.1287877

**Published:** 2024-02-08

**Authors:** Julio Cesar Cavalcanti, Ronaldo Rodrigues da Silva, Anders Eriksson, Plinio A. Barbosa

**Affiliations:** ^1^Laboratory of Phonetics, Department of Linguistics, Stockholm University, Stockholm, Sweden; ^2^Integrated Acoustic Analysis and Cognition Laboratory, Pontifical Catholic University of São Paulo, São Paulo, Brazil; ^3^Department of Linguistics, Institute of Language Studies, University of Campinas, Campinas, Brazil; ^4^National Institute of Criminalistics, Federal Police of Brazil, Brasília, Brazil

**Keywords:** speech analysis, phonetics, acoustic-phonetics, forensic phonetics, automatic speaker recognition

## Abstract

This study assessed the influence of speaker similarity and sample length on the performance of an automatic speaker recognition (ASR) system utilizing the SpeechBrain toolkit. The dataset comprised recordings from 20 male identical twin speakers engaged in spontaneous dialogues and interviews. Performance evaluations involved comparing identical twins, all speakers in the dataset (including twin pairs), and all speakers excluding twin pairs. Speech samples, ranging from 5 to 30 s, underwent assessment based on equal error rates (EER) and Log cost-likelihood ratios (Cllr). Results highlight the substantial challenge posed by identical twins to the ASR system, leading to a decrease in overall speaker recognition accuracy. Furthermore, analyses based on longer speech samples outperformed those using shorter samples. As sample size increased, standard deviation values for both intra and inter-speaker similarity scores decreased, indicating reduced variability in estimating speaker similarity/dissimilarity levels in longer speech stretches compared to shorter ones. The study also uncovered varying degrees of likeness among identical twins, with certain pairs presenting a greater challenge for ASR systems. These outcomes align with prior research and are discussed within the context of relevant literature.

## 1 Introduction

### 1.1 Automatic speaker recognition through neural network

Neural network analysis is a machine learning technique that is capable of modeling highly complex non-linear relationships in data, namely, when changes in one variable are not directly proportional to the changes in another variable. Through a process known as training, neural networks can learn complex patterns and relationships in data, where the weights of the connections between neurons are adjusted based on the input and desired output (LeCun et al., 1998; Domingos, 2012; Jurafsky and Martin, 2023).

Models based on neural networks have various applications in science, spanning from image recognition, speech recognition, and natural language processing, among others. Its key advantage is the ability to learn from complex datasets and to generalize their learning to new, unseen data. Such ability makes them highly effective for tasks that involve pattern recognition or classification (LeCun et al., 2015).

Different studies have applied neural network-based analyses with speech data to address a range of goals, such as automatic speech recognition (Graves and Jaitly, 2014; Wu et al., 2020), automatic speaker recognition (Devi et al., 2020), speech emotion recognition based on audio (Jiang et al., 2019) and audio-visual information (Hussain et al., 2022), speech enhancement for cochlear implants—aiming to improve the clarity and quality of speech comprehension for individuals with hearing loss (Kang et al., 2021), and, the assessment of speech/voice impairments in chronic degenerative disorders (Maskeliūnas et al., 2022).

Concerning the first two tasks mentioned earlier, namely automatic speech and speaker recognition, it is worth noting that the acronym ASR is widely employed in both tasks. Nevertheless, the distinction in their contexts plays a crucial role in separate technological domains. On the one hand, ASR in the context of Automatic Speech Recognition regards a technology crafted for converting spoken language into written text. On the other hand, ASR in Automatic Speaker Recognition concerns a specialized field centered on comparing individuals through their distinctive vocal characteristics. For the purposes of the present study, it is essential to clarify that the term ASR will specifically pertain to the latter task—Automatic Speaker Recognition.

In the field of Automatic Speaker Recognition (henceforth, ASR), the Gaussian Mixture Model-Universal Background Model (GMM-UBM) has been a widely used technique for several years. This approach models the spectral characteristics of a speaker's voice. It involves analyzing the statistical distribution of acoustic features, including Mel-Frequency Cepstral Coefficients (MFCCs) and filterbank energies, which are representations of the energy distribution across different frequency bands in an audio signal. The goal of the GMM-UBM method is to discern speaker-specific characteristics that are crucial for distinguishing between speakers.

The GMM-UBM has been shown to be effective in ASR, achieving high recognition rates on text-independent data sets. For instance, in Reynolds and Rose (1995), Gaussian mixture-based speaker model attained 96.8% recognition accuracy using 5-s clean speech utterances and 80.8% accuracy using 15-s telephone speech utterances with a 49-speaker population.

GMM-UBM i-vector, which was proposed to be used in speaker verification by Dehak et al. (2011), has become the state-of-the-art technique in the speaker verification field. It has a compact representation of speaker characteristics derived from speech signals. It is generated through a two-step process involving a Universal Background Model (UBM) and a Total Variability Matrix.

Despite its effectiveness, GMM has been replaced by newer and more advanced approaches that are able to capture more complex patterns and contextual information, leading to improved accuracy and robustness, such as deep neural network-based (DNN) models like recurrent neural networks (RNNs) and convolutional neural networks (CNNs).

More recently, a feature extraction method based on neural networks, called *x*-vector (Snyder et al., 2018), has been developed primarily for ASR. It is based on a deep neural network (DNN) architecture with multiple fully connected layers featuring a temporal context (referred to as “frames”) in each layer. Due to the broader temporal context, the architecture is termed Time-Delay Neural Network (TDNN).

In a multi-laboratory evaluation of forensic voice comparison systems, conducted under conditions that closely resemble those encountered in real forensic cases (Morrison and Enzinger, 2019), deep neural network (DNN) systems outperformed Gaussian mixture model (GMM) approaches. The top-performing GMM system, based on i-vector, achieved an equal error rate (EER) of 7.0%. However, the DNN system, utilizing *x*-vector, achieved a significantly lower EER of 2.2%.

Lately, the approach based on Emphasized Channel Attention, Propagation and Aggregation in Time Delay Neural Network (ECAPA-TDNN) (Desplanques et al., 2020) available in SpeechBrain, which combines the benefits of convolutional and recurrent neural networks, was found to outperform both the *x*-vector and the ResNet-34 (Zeinali et al., 2019) by a large margin in text-independent speaker verification tasks (Ravanelli et al., 2021), i.e., without requiring specific spoken text or prompts from the speakers, only selecting stretches of audio recordings. In addition, ASR technologies based on deep neural network (DNN) models also improve the systems' ability to handle irrelevant/contextual input variations, focusing on representations that are selective to the aspects of a signal that are important for discrimination while invariant to other aspects (LeCun et al., 2015).

However, it is worth noting that DNN systems are not invariant to relevant aspects, especially those with implications to the forensic speaker comparison practice, such as the duration of audio samples. According to Sztahó et al. (2023), while using the ECAPA-TDNN model, which is employed in the present study, the system's performance tended to improve as the sample durations increased. Conversely, the study found that the ASR system's performance does not appear to be affected by mismatched language during likelihood ratio (LR) calibration between the model and the corpus.

Despite the considerable advances in the past few years, our understanding of the impact of essential factors, such as the level of similarity across speakers, on the performance of ASR systems is still limited, particularly when considering more recent approaches. In the following, we briefly review some studies regarding ASR with similar (genetically related) subjects.

### 1.2 Speaker recognition studies with twin speakers

It is widely understood that ASR systems face significant challenges when contrasting identical twins' voices. The main reason is that these systems rely on identifying the unique acoustic characteristics of an individual's voice—like the ones we mentioned before. However, since identical twins share striking similar genetic makeup and sources of environmental exposure, their speech characteristics can be challenging to differentiate for ASR systems. Acoustic-phonetic analyses of identical twin's speech, as those based on melodic, spectral, and timing parameters, have consistently revealed a remarkable degree of acoustic similarity, surpassing that observed in unrelated individuals (Fernández and Künzel, 2015; Sabatier et al., 2019; Cavalcanti et al., 2021a,b, 2022).

As a result of such a higher level of acoustic-phonetic similarities, ASR systems can produce errors and reduced accuracy in speaker recognition, particularly in fields like law enforcement and security, where speaker recognition is widely used for authentication purposes. Unarguably, these errors can have serious consequences, making it crucial to address this challenge and develop more effective solutions to improve speaker recognition systems' reliability and accuracy in differentiating not only related subjects, but also similar-sounding voices.

Ariyaeeinia et al. (2008) carried out a study aiming to assess speaker verification technology's ability to discriminate between identical twins. The data consisted of 49 pairs of identical twins (40 pairs of females and nine pairs of males). Two token recordings were collected for each speaker. The first token was a poem around 60 s long. The second token was the individual's birth date, spoken as digits, around 5 s in duration. The tests were conducted using the “short” and “long” test tokens. For every test, the results were obtained using the GMM-UBM scoring procedure and used as the baseline results. The scores obtained this way were then subjected to unconstrained cohort normalization (UCN) based on a cohort size of three—a technique used in speaker recognition systems for score normalization. It adjusts the recognition scores by comparing them against a cohort of speakers, indirectly helping to account for variability in the scores that may arise from factors like recording conditions and background noise.

The authors of that study observed that long utterances led to lower error rates. Regarding twin comparisons, the EERs are about 10 and 5% in short and long test tokens, respectively. This clearly indicates the non-genetic (extraneous) factors influencing the characteristics of the voices of each pair of twins. The use of UCN was found to reduce the EERs significantly. According to the authors, the results seem to agree with the suggested capability of UCN to reduce the impostor scores in relation to those of true speakers. As mentioned by the authors, UCN exploits the non-genetic characteristics of the twins' voices to enhance the discrimination capability of ASR.

Künzel (2010) performed an experiment involving nine male and 26 female pairs of identical twins. Each twin produced one read and one spontaneous speech sample, which were used to calculate inter-speaker, intra-twin pair, and intra-speaker likelihood ratio (LR) distributions using an automatic Bayesian-based system for forensic speaker comparison called Batvox (version 3.1).

The results showed that under certain conditions, the ASR system could distinguish even the vast majority of very similar-sounding voices, such as those of identical twins. However, the system's performance was found to be superior for males compared to female voices. Overall, that study demonstrated the potential of ASR systems to distinguish between even very similar-sounding voices, such as those of identical twins. However, the results also suggested that sex-related differences may affect the system's performance.

In their study, Fernández and Künzel (2015) evaluated the performance of Batvox (Version 4.1) using a sample of 54 Spanish speakers. The sample included 24 monozygotic (MZ) twins, 10 dizygotic (DZ) twins, eight non-twin siblings (B), and 12 unrelated speakers between the ages of 18 and 52, all of whom spoke Standard Peninsular Spanish.

The main hypothesis was that the cepstral features of the system, which depend largely on anatomical-physiological variables, would be gene-dependent. Therefore, a higher similarity in MZ twins would be expected in comparison to the other groups of speakers. The results supported the hypothesis, showing a decreasing scale of similarity coefficients: MZ > DZ > B > US. This pattern corresponded precisely with the decreasing kinship degree of the four speaker groups. The authors suggested that the features of the ASR system are largely genetically conditioned, making it useful and robust for comparing speech samples of known and unknown origin.

In their study, Sabatier et al. (2019) conducted verification experiments with 167 pairs of twins, using various train-test conditions and durations. Participants were asked to read their random identification number aloud and then the rainbow passage, followed by an interview portion where they answered pre-determined questions about themselves. The scripted speech was ~30 s, while the unscripted speech was ~70 and 180 s long, recorded on two different occasions. The GMM-UBM method was used as the reference method for voice matching.

Results showed that standard voice matching algorithms had more difficulty distinguishing between identical twins than non-related individuals, as evidenced by consistently lower accuracy based on comparisons of the equal error rate (EER) from a similarly collected non-twins database. Female speakers were found to be more difficult to distinguish than their male counterparts, and conversational data was associated with poorer twin differentiation due to higher intra-speaker variability. The same trend was observed for non-twin subjects.

While previous studies have explored the intersection of ASR systems and identical twin speech, it is worth emphasizing the continued relevance of research in this domain. Notably, studies in this area often exhibit variations in methodological aspects, including the characteristics of the ASR system employed, the attributes of the speech material under analysis, and the number of speakers considered.

The present study adds to the body of knowledge regarding ASR research on twins while adding to the linguistic diversity in ASR research by focusing on Brazilian Portuguese speakers. Engaging in research across diverse speaker groups allows us to develop a more comprehensive knowledge base. The methodological design adopted in this research not only contributes to grasping the distinct difficulties associated with comparing between similar speakers but also facilitates quantifying these challenges in light of contemporary ASR models.

## 2 Materials and method

The present study, registered under protocol 95127418.7.0000.8142, was evaluated and approved by the ethical committee at Campinas State University (UNICAMP). All participants voluntarily agreed to be part of the research verbally and by signing a participant consent form.

### 2.1 Participants

The participants were 20 speakers comprising 10 identical male twin pairs, all of them Brazilian Portuguese (BP) speakers from the same dialectal area. The participants' age ranged between 19 and 35 years, with a mean of 26.4 years. All identical twin pairs were codified with letters and numbers, such as A1, A2, B1, B2, C1, C2, D1, D2, and so on. The same letters indicate that the speakers are identical twins and, therefore, related.

Regarding the comparison of non-genetically related participants, this was achieved using a cross-pair comparison approach (e.g., A1 × B1; A1 × B2; A2 × B1, A2 × B2, and so on). Overall, 190 speaker pair combinations were carried out for comparisons involving all speakers (including twins), and 180 pair combinations for comparisons of non-genetically related speakers (excluding twins).

It is important to highlight that twin speakers constitute only about 5% of the total number of speakers in the data set for comparisons encompassing all participants, including non-twin pairs. Despite being a relatively small subset, the inclusion of twin speech data is anticipated to introduce a greater level of complexity in the task of speaker discrimination. This addition is likely to make the process of distinguishing between speakers more challenging.

The inclusion of only male participants in this study is attributed to the reliance on a pre-existing database (Cavalcanti, 2021). The emphasis on male speakers in this database was designed to mirror the gender distribution observed in specific categories of crimes, where a substantial proportion of suspects or persons of interest tend to be male.

### 2.2 Recordings

All recordings were made with a sample rate of 44.1 kHz and 16-bit, using an external audio card (Focusrite Scarlett 2i2) and two headset condenser microphones (DPA 4066-B). The recordings were undertaken in silent rooms in the cities where the speakers resided. The recordings were made in two different sessions, as described below. Approximately 5–10 min of unedited conversational speech (dialogue) and 3–5 min of unedited interview speech were available per speaker.

#### 2.2.1 Session I

In the first recording session, the speech materials comprised spontaneous mobile smartphone conversations (dialogues) between twin brothers who were very familiar with each other. The participants themselves decided the topics of discussion.

The recording took place with the speakers in separate rooms, communicating via mobile phones without visual contact. The speakers were prompted to initiate the conversation using mobile phones while high-quality microphones recorded the exchange. The resulting unedited and unfiltered audio signals were processed and registered in two channels, preserving their acoustic characteristics.

#### 2.2.2 Session II

During the second recording session, the researcher interviewed the speakers and asked them to describe their daily routine, from waking up to going to bed and their leisure activities. Following this, the participants were asked to describe their routines from previous weeks, including a week, a month, and a year ago, and how their routines had changed over the course of the year.

Besides the fact that a different speaking style was elicited, an important aspect of this step was the intentional reduction of familiarity between the interviewer and the participants, which aimed to simulate the setting of forensic speaker comparison, where an unfamiliar interviewer questions individuals.

### 2.3 Analysis

In the present study, we applied the SpeechBrain toolkit for ASR analyses. The SpeechBrain toolkit is an open-source, comprehensive solution for speech processing tasks. Its primary objective is to enable research and development of neural network-based speech technologies through its flexible, user-friendly, and well-documented design (Ravanelli et al., 2021).

ASR using SpeechBrain refers to applying machine learning algorithms, namely, neural networks. The system was evaluated by obtaining the LR scores using logistic regression based on scikit-learn (Pedregosa et al., 2011) package adopting a cross-validation technique using two different methods: the leave-one-speaker-out and leave-two-speaker-out methods.

The cross-validation using the leave-one-speaker-out method was applied for testing recordings of the same speakers (intra-speaker) but in varying speaking styles, i.e., interview vs. dialogue. This approach involved using each speaker's recording as a test case once, with the recordings from all other speakers forming the training set. Additionally, the leave-two-speaker-out method was applied to assess recordings from different speaker pairs (inter-speakers). In this scenario, each time a pair of speakers was tested, recordings from all remaining speakers in the database were used to create the training set.

A high-level overview of the steps adopted in the analyses is presented in the following:

- The wave PCM format recordings from the twin siblings were resampled at 16 kHz in order to adapt them to the neural network model used.
- Two audio recordings were selected, each containing 60 s of net speech from each of the siblings, one from Session I and another from Session II, totaling 40 recordings.
- Each of these recordings was divided into samples of 5, 10, 15, 20, and 30 s, resulting in 12 samples of 5 s, six samples of 10 s, four samples of 15 s, three samples of 20 s, and two samples of 30 s per speaker.
- All of these audio samples were processed by the ECAPA-TDNN model (Desplanques et al., 2020) provided by the Speechbrain project (Desplanques et al., 2021), which was pre-trained using the training data from the Voxceleb1 (Nagrani et al., 2017) and Voxceleb2 (Chung et al., 2018) databases, obtaining a 192-dimensional embedding vector for each sample.
- Specifically for assessing the level of similarity between twin speakers, mean and standard deviation similarity scores were computed as a function of twin pairs and sample sizes. The cosine similarity was used to calculate the level of similarity between the embedding vectors of each audio pair, which ranges from −1 to +1. Here, a value of +1 indicates identical feature vectors, a value of 0 indicates orthogonal vectors (no similarity), and a value of −1, though rare in this context, would indicate completely dissimilar vectors. The similarity or even dissimilarity between the audios are based on the acoustic features judged relevant by the model. It's worth noting that the similarity measure is based on the feature vectors extracted from the audio, not the audio themselves.
- After computing all the scores between the speakers in all scenarios, the system was assessed using two discriminatory performance metrics: Equal Error Rate (EER) (Equation 1) and log-likelihood-ratio cost (Cllr) (Equation 2), as outlined below.

The EER is a commonly used metric in speaker recognition tasks and is defined as the point at which the false acceptance rate (FAR) and false rejection rate (FRR) are equal (Conrad et al., 2012). EER is calculated through the Equation (1):


(1) $$\begin{array}{l} EER=\frac{1}{2}\left(\frac{FAR+FRR}{2}\right) \end{array}$$

In Equation (1), The FAR is the rate at which the system incorrectly accepts an impostor as the target speaker, while the FRR is the rate at which the system rejects the target speaker incorrectly. By computing the EER, we can determine the point at which the system is equally likely to make both types of errors.

The Log-likelihood-ratio cost (Cllr) is an empirical estimate of the precision of likelihood ratios proposed by Brümmer and Du Preez (2006). According to Morrison et al. (2011), such an estimate has the desired properties of being based on likelihood ratios, being continuous, and more heavily penalizing worse results (i.e., providing less support for the consistent-with-fact hypothesis or more support for the contrary-to-fact hypothesis). Cllr is calculated through the Equation (2):


(2) $$\begin{array}{l} C_{llr}=\frac{1}{2}\left(\frac{1}{N_{ss}}\overset{N_{ss}}{\underset{i=1}{\sum }}\log_{2}\left[1+\frac{1}{LR_{{ss}_{i}}}\right]+\frac{1}{N_{ds}}\overset{N_{ds}}{\underset{j=1}{\sum }}\log_{2}\left[1+LR_{{ds}_{j}}\right]\right) \end{array}$$

In Equation (2), *Nss* and *Nds* are the number of same-speaker and different-speaker comparisons, respectively, whereas *LRss* and *LRds* are the likelihood ratios derived from same-speaker and different-speaker comparisons, respectively. A same-origin penalty value is *log*_2_(1+1/*LRs*), and a different-origin penalty value is *log*_2_(1+*LRd*).

Probabilistic Linear Discriminant Analysis (PLDA) is commonly used in ASR systems for modeling speaker and channel variability distributions. It generally benefits from multiple recordings per speaker, aiding the model in capturing the nuanced variations in a speaker's voice. However, the existing database has only one recording per speaker for each analyzed speaking style. Given this constraint, the decision was made to employ cosine similarity, as it allows for comparisons between single samples.

#### 2.3.1 Speaker comparisons

ASR analyses were applied to three types of speaker comparisons. The first concerns comparisons performed between speakers of the same twin pair (i.e., intra-twin pair), the second regards comparisons among all subjects in the study, twins and non-twins, and the third comprises comparisons of non-twin speakers only (i.e., unrelated speakers).

It is worth noting that, in terms of restricted population characteristics, the last type of speaker comparison is the closest to what may be observed in real-life scenarios, given that the twin population is over-represented in the second type. However, the first and second comparison settings serve experimental ends, namely, understanding how ASR systems perform in challenging adverse conditions. In this regard, by examining both realistic and controlled conditions, researchers can develop and refine ASR technologies to achieve more accurate and reliable results.

## 3 Results

Tables 1–3 present the results of the ASR performances as a function of speech sample length in three different-speakers (DS) comparison scenarios. The same-speaker (SS) and different-speaker (DS) tests were conducted using data from distinct speaking styles: interview and spontaneous dialogue.

**Table 1:** System performance limiting DS comparisons to twin siblings.

**Samples**	**EER**	**Cllr**
12 × 5 s	0.20	0.63
6 × 10 s	0.17	0.52
4 x × 15 s	0.16	0.47
3 × 20 s	0.15	0.44
2 × 30 s	0.10	0.45

**Table 2:** System performance considering the comparison of all pairs of DS in the study, including comparisons between twin pairs.

**Samples**	**EER**	**Cllr**
12 × 5 s	0.02	0.08
6 × 10 s	0.01	0.05
4 × 15 s	0.01	0.05
3 × 20 s	0.01	0.04
2 × 30 s	0.00	0.04

**Table 3:** System performance considering the comparison of all speakers in the study, excluding comparisons between twin pairs.

**Samples**	**EER**	**Cllr**
12 × 5 s	0.01	0.03
6 × 10 s	0.00	0.01
4 × 15 s	0.00	0.01
3 × 20 s	0.00	0.01
2 × 30 s	0.00	0.01

Table 1 depicts the system performance obtained from analyzing data from identical twins through SS and DS limited to twin siblings. Table 2 shows system performance when the comparisons were made among all speakers in the corpus, including twin speakers in DS comparisons. Finally, Table 3 displays the system performance considering comparisons carried out across all subjects, excluding identical twins in DS comparisons.

**Figure 1:**
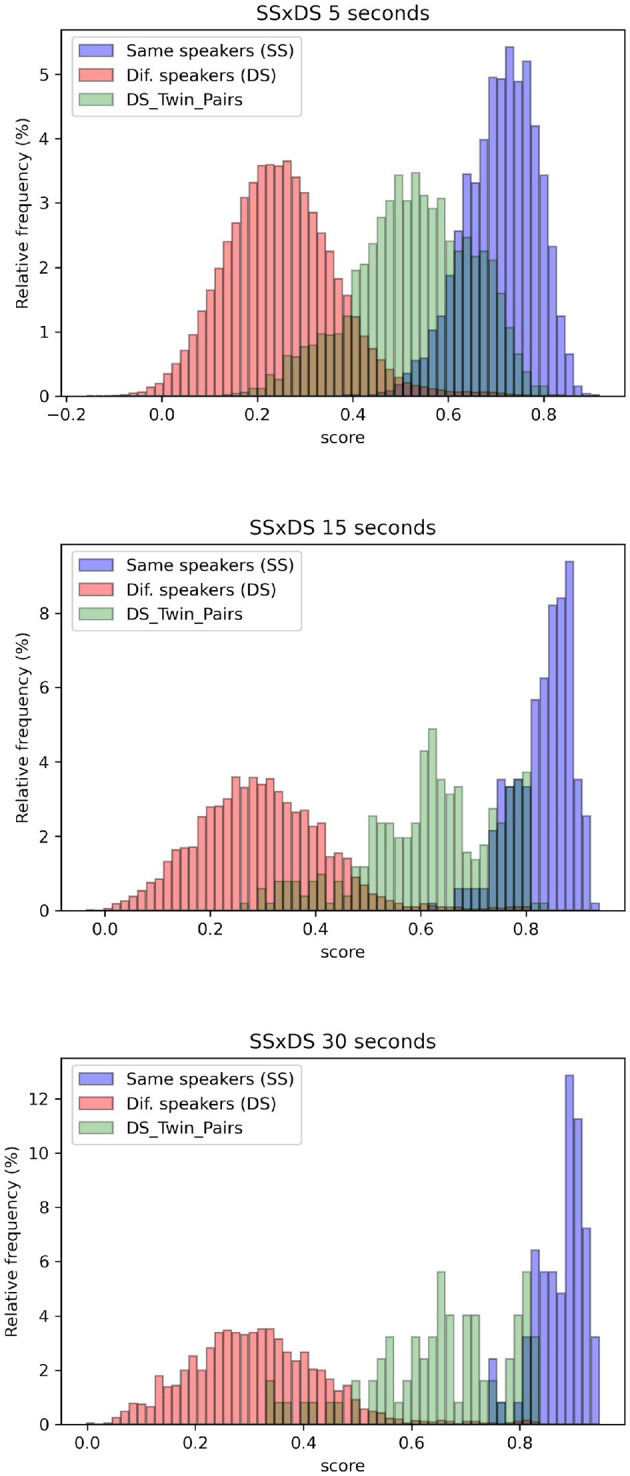
Blue histograms depict the distribution of scores obtained in SS comparisons, while the red histograms represent scores from DS comparisons for all speakers, including twin pairs. Additionally, the green histograms specifically represent DS scores for identical twin pairs, for samples of 5, 15, and 30 s long.

The distributions of scores obtained in SS and DS comparisons are visually represented in Figure 1 for samples of 5, 15, and 30 s duration. The histograms depicting scores for SS comparisons are presented in blue, while those for DS comparisons encompassing all subjects, including twin speakers, are depicted in red. Additionally, histograms displaying scores solely from DS comparisons involving identical twin pairs are shown in green. It is possible to observe considerable overlap between the blue histograms (SS) and green histograms (DS from comparisons between twin siblings), illustrating higher EER and Cllr for this scenario.

**Figure 2:**
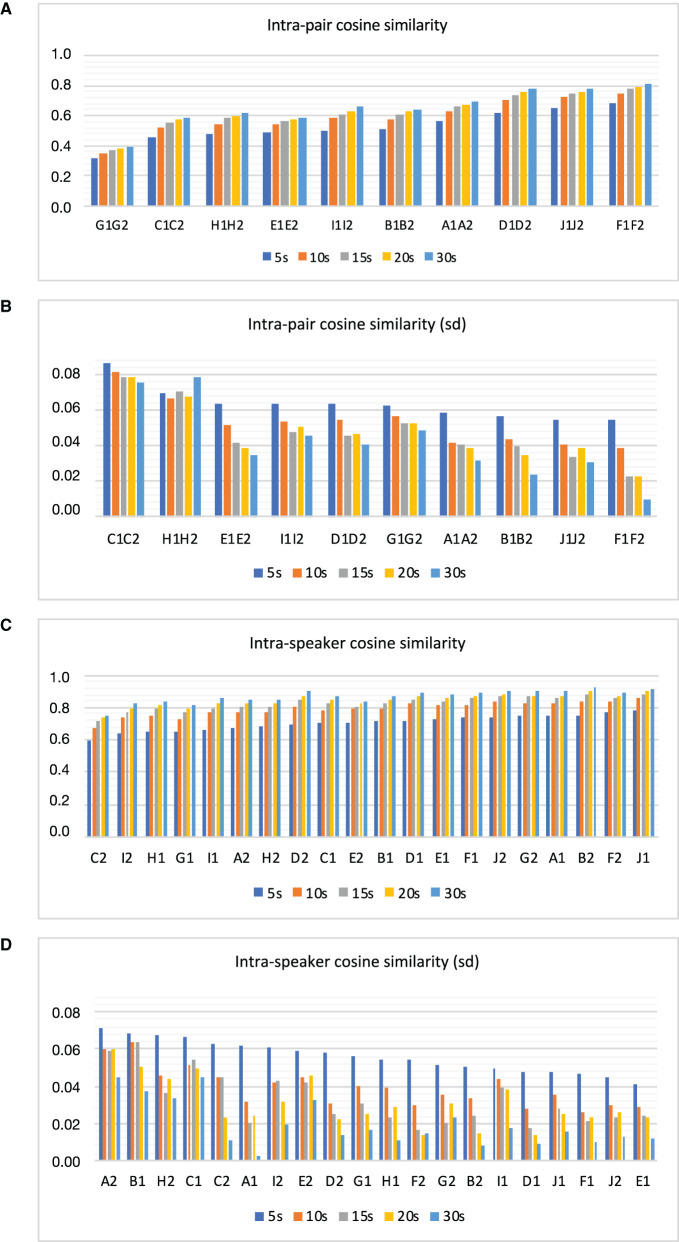
Intra-pair cosine similarity **(A)**, standard deviation of intra-pair cosine similarity **(B)**, intra-speaker cosine similarity **(C)**, and standard deviation of intra-speaker cosine similarity **(D)** as a function of sample size.

Moreover, Figures 2A, B visually depict the results related to the analysis of intra-twin pair cosine similarity based on sample size. Figure 2A shows the mean intra-twin pair cosine similarity in ascending order, while Figure 2B illustrates their respective standard deviations in descending order. For a more detailed numerical evaluation of these figures, please refer to Table 4.

**Table 4:** Mean and standard deviation (SD) values of cosine similarity for intra-identical twin pair (different-speakers) comparisons as a function of sample sizes in increasing order of similarity—ordered by the first numeric column (5 s).

	**Sample size**									
	**5 s**		**10 s**		**15 s**		**20 s**		**30 s**	
**Twin pair**	**Mean**	**SD**	**Mean**	**SD**	**Mean**	**SD**	**Mean**	**SD**	**Mean**	**SD**
G1-G2	0.31	0.06	0.35	0.06	0.37	0.05	0.38	0.05	0.39	0.05
C1-C2	0.46	0.09	0.52	0.08	0.55	0.08	0.57	0.08	0.58	0.08
H1-H2	0.47	0.07	0.55	0.07	0.58	0.07	0.59	0.07	0.62	0.08
E1-E2	0.49	0.06	0.54	0.05	0.56	0.04	0.57	0.04	0.59	0.04
I1-I2	0.50	0.06	0.59	0.05	0.61	0.05	0.63	0.05	0.66	0.05
B1-B2	0.51	0.06	0.58	0.04	0.60	0.04	0.62	0.03	0.64	0.02
A1-A2	0.57	0.06	0.63	0.04	0.66	0.04	0.67	0.04	0.69	0.03
D1-D2	0.61	0.06	0.71	0.05	0.74	0.04	0.75	0.05	0.77	0.04
J1-J2	0.65	0.06	0.72	0.04	0.75	0.03	0.76	0.04	0.78	0.03
F1-F2	0.69	0.05	0.75	0.04	0.78	0.02	0.79	0.02	0.81	0.01

In addition, Figures 2C, D visually represent the results concerning the analysis of intra-speaker cosine similarity based on sample size. Figure 2C presents the mean intra-speaker cosine similarity in ascending order, while Figure 2D illustrates their respective standard deviations in descending order. For a more in-depth numerical assessment of these figures, see Table 5.

**Table 5:** Mean and standard deviation (SD) values of cosine similarity for intra-speaker (same-speaker) comparisons as a function of sample sizes in increasing order of similarity—ordered by the first numeric column (5 s).

	**Sample size**									
	**5 s**		**10 s**		**15 s**		**20 s**		**30 s**	
**Speaker**	**Mean**	**SD**	**Mean**	**SD**	**Mean**	**SD**	**Mean**	**SD**	**Mean**	**SD**
C2	0.59	0.06	0.67	0.05	0.72	0.05	0.74	0.02	0.75	0.01
I2	0.64	0.06	0.74	0.04	0.77	0.04	0.80	0.03	0.83	0.02
H1	0.65	0.06	0.74	0.04	0.79	0.02	0.82	0.03	0.84	0.01
G1	0.65	0.06	0.73	0.04	0.77	0.03	0.79	0.02	0.82	0.02
I1	0.66	0.05	0.77	0.04	0.79	0.04	0.83	0.04	0.86	0.02
A2	0.68	0.07	0.77	0.06	0.81	0.06	0.83	0.06	0.85	0.05
H2	0.68	0.07	0.77	0.05	0.80	0.04	0.83	0.04	0.85	0.03
D2	0.70	0.06	0.81	0.03	0.85	0.03	0.87	0.02	0.90	0.01
C1	0.71	0.07	0.78	0.05	0.82	0.05	0.84	0.05	0.87	0.05
E2	0.71	0.06	0.79	0.05	0.81	0.04	0.83	0.05	0.84	0.03
B1	0.71	0.07	0.79	0.06	0.83	0.06	0.85	0.05	0.87	0.04
D1	0.72	0.05	0.82	0.03	0.85	0.02	0.87	0.01	0.89	0.01
E1	0.72	0.04	0.81	0.03	0.84	0.02	0.86	0.02	0.88	0.01
F1	0.74	0.05	0.82	0.03	0.86	0.02	0.87	0.02	0.89	0.01
J2	0.74	0.04	0.83	0.03	0.87	0.02	0.88	0.03	0.90	0.01
G2	0.74	0.05	0.82	0.04	0.86	0.02	0.87	0.03	0.90	0.02
A1	0.75	0.06	0.83	0.03	0.86	0.02	0.87	0.02	0.90	0.00
B2	0.75	0.05	0.84	0.03	0.88	0.02	0.90	0.02	0.92	0.01
F2	0.77	0.05	0.83	0.03	0.86	0.02	0.87	0.01	0.90	0.02
J1	0.78	0.05	0.86	0.04	0.88	0.03	0.90	0.03	0.92	0.02

Note that sorting the similarity scores in ascending order in Figure 2A provides insights into the degree of likeness or dissimilarity among pairs. This allows us to evaluate which acoustic parameters related to voice quality could effectively elucidate this dissimilarity in future studies. Conversely, organizing the standard deviation values of similarity scores in Figure 2B in descending order provides insights into the degree of variability observed in the similarity estimation. This ordering spans from pairs with the highest variability to those with the lowest. The same reasoning applies to the assessment of intra-speaker (same-speaker) cosine similarity in Figures 2C, D.

A common trend depicted in Tables 1–3 regards the observation that longer speech samples yielded better performances, as suggested by the comparison of EER and Cllr values. However, after a certain point, the level of improvement for Cllr appeared to approach stabilization between the 10 and 15 s threshold.

When comparing Tables 1, 2, it becomes apparent that the system faced a considerable challenge when processing data from twin speakers, as opposed to when data from all speakers was used. In the case of twin speakers, the system's performance ranged from 10% (0.10) to 19% (0.20) EER and from 0.45 to 0.63 Cllr. However, when comparing all subjects, including twin pairs, the system's performance ranged from practically 0% (0.004) to 1% (0.016) EER and from 0.04 to 0.08 Cllr.

Figure 1 provides a graphical representation of how the system's performance is influenced by the attributes of the individuals being compared. One can observe that the red histograms—which correspond to comparisons made across all speakers—are the farthest from the blue histograms, representing the distributions of comparisons made between the same speaker. In contrast, the green histograms—which represent comparisons between twin pairs—are the closest to the blue histograms, indicating the utmost degree of challenge when distinguishing between them.

It is worth noting that removing twin speakers from the list of DS comparisons had a minor effect on the EER and Cllr values, as shown in Table 3. The impact was characterized by a slight decrease in already low EER and Cllr values, resulting in even lower scores. Comparing Tables 2, 3, it was evident that EER values were below 1% for all testing conditions when no twin speakers were included, whereas when they were included, EER values were below 2%.

Regarding the similarity/dissimilarity levels observed between identical twin pairs as presented in Table 4, the high cosine similarity scores are unsurprising and suggest that twin speakers are more similar than dissimilar. As may be recalled, these scores are based on cosine similarity, which ranges from −1 to +1. Here, a value of +1 indicates identical feature vectors, a value of 0 indicates orthogonal vectors (no similarity), and a value of −1, though rare in this context, would indicate completely dissimilar vectors.

However, the results depicted in Figure 2A and summarized in Table 4 suggest that some twin pairs were more similar than others regarding their acoustic properties. As can be seen, across all pairs, G1–G2 was found to be the most dissimilar one, while F1–F2 was the twin pair presenting the highest level of similarity as reflected in their intra-pair cosine similarity scores across various sample sizes.

Furthermore, examining standard deviation values in Figure 2B and Table 4 across various sample sizes reveals a distinct pattern. There is a noticeable trend of lower standard deviation values associated with larger samples as opposed to smaller ones. This tendency becomes more evident when comparing 5 and 30 s intervals, indicating reduced variability in the estimation of speaker similarity/dissimilarity levels over longer durations of speech compared to shorter ones.

Upon examining Figure 2A, it becomes evident that with an increase in sample size, twin pairs exhibit a slight increase in dissimilarity, as indicated by a numerical rise in similarity scores from 5 s to 30 s samples. Regarding standard deviation values in Figure 2B, as previously noted, larger speech sample sizes tended to exhibit reduced variability in estimating similarity scores.

A similar pattern is observed in the examination of intra-speaker similarity. As illustrated in Figure 2C, intra-speaker similarity values surpass those of inter-speaker similarity depicted in Figure 2A. Furthermore, these values escalate with an augmentation in sample size, aligning with heightened same-speaker similarity. Additionally, larger speech sample sizes tend to demonstrate a notable decrease in variability associated with the assessment of same-speaker similarity. Tables 4, 5 allow a detailed numeric comparison of such trends.

## 4 Discussion

In this current study, the duration of speech samples has been demonstrated to influence the performance of Automatic Speech Recognition (ASR). This outcome is compatible with previous findings presented in the literature, as they suggest an advantage in performance for longer speech samples in comparison to shorter (Gelfer et al., 1989; Reynolds and Rose, 1995; Sztahó et al., 2023).

For instance, a study by Reynolds and Rose (1995) found that a GMM speaker comparison model attained 96.8% accuracy using 5-s clean speech utterances and 80.8% accuracy using 15-s telephone speech utterances. ASR performance also seemed to improve with increasing training data. The largest increase in performance occurred when the utterance length increased from 30 to 60 s. Increasing the utterance length to 60 s improved the performance only marginally. The outcomes of that study suggest that longer speech samples are particularly important when dealing with lower audio-quality data.

A study by Gelfer et al. (1989) found that speech samples of 10 s or more are generally needed for accurate speaker recognition. However, they also showed that recognition rates could be improved with longer speech samples. Moreover, a recent study by Sztahó et al. (2023), using the same ECAPA-TDNN model employed in this study, showed that the performance tends to improve as the duration of the sample increases, and in the best scenario, samples of 10 s duration achieved an EER of 0.2%.

The impact of speech sample duration on ASR performance varies based on factors like signal quality, system complexity, and speaker characteristics (Künzel, 2010; Poddar et al., 2018; Sabatier et al., 2019). Generally, longer speech samples in ASR systems yield higher accuracy, capturing comprehensive information about voice characteristics and speech patterns. Longer speech stretches also appear to allow the stabilization of internal variation, reducing uncertainty in speaker comparison, especially in mismatched speaking styles. Conversely, shorter speech samples show more pronounced variation, increasing uncertainty in voice comparisons. Research on the temporal stability of acoustic-phonetic parameters, such as fundamental frequency, suggests stabilization within around 30 s of recorded speech, with a median time of ~10 s (Arantes and Eriksson, 2014).

A crucial point to note in the context of the present study is that, as the sample size increased, twin pairs showed a slight increase in similarity, seemingly conflicting with the overall improvement in the discriminatory system's performance. However, a plausible explanation lies in the simultaneous rise in intra-pair similarity, which corresponds with an increase in already higher intra-speaker similarity scores. The conjunction of heightened intra-speaker similarity and a reduced standard deviation associated with its calculation may provide insight into this outcome.

It should be noted that the benefit of longer speech samples on accuracy is expected to reach a certain limit where it tends to stabilize. In the current study, when comparing Cllr scores in Tables 1–3, very similar results were obtained for speech samples that were 15, 20, or 30 s long. However, there was a more significant difference between the 5- and 30-s speech samples. This suggests that while longer speech samples can provide a considerable improvement in accuracy, there may be a point where additional speech information is not as useful. Based on the specific characteristics of the data used in this study (i.e., high-quality recordings), a threshold between 10–15 s seems promising. However, further analyses that take into consideration various speaker groups are necessary.

The observed slight enhancement in performance, achieved by excluding identical twins' speech data, aligns with expectations, given the presumption of greater similarity in twins' voices compared to unrelated speakers. Acoustic-phonetic analyses conducted on the same speaker group, as addressed in this study, have consistently demonstrated significantly heightened acoustic similarity among identical twin speakers in comparison to unrelated individuals, cf. Cavalcanti et al. (2021a,b, 2022).

While the research by Cavalcanti et al. (2021a,b, 2022, 2023) predominantly focused on analyzing male voices, limiting broader generalizations, studies incorporating more representative samples of both sexes suggest that the elevated similarity among twin subjects, in contrast to unrelated subjects, is not exclusive to a specific gender group (cf. Przybyla et al., 1992; Nolan and Oh, 1996; Van Lierde et al., 2005; Weirich, 2012). Despite the potential influence of sex on the difficulty of differentiating twin pairs, as proposed by Sabatier et al. (2019), the overall trend of increased similarity extends across gender boundaries.

Including twin voices in the ASR task may increase the system's complexity and potentially challenge its performance. This can result in lower performance metrics, such as higher error rates and greater Cllr. Nevertheless, this practice can be advantageous in some experimental settings, as it can lead to less optimistic outcomes, especially when dealing with high-quality recordings that may not represent the reality of real-world forensic practice. Researchers can obtain a more comprehensive understanding of the system's performance under diverse conditions by intentionally introducing challenging factors into the analysis, such as twin voices.

As pointed out by Sabatier et al. (2019), the issue of speaker variability becomes more pronounced when the number of speakers in the evaluation set increases, as there is a higher probability that two voices may sound more alike. The differentiation of intra-twin pairs' voices can benefit general ASR systems because the inclusion of their voices in the training procedure can simulate such an effect. This is because their shared voice similarities stretch the limits of the potential inter-speaker similarities one would expect in a large speaker database.

The observation that certain twin pairs exhibit varying degrees of similarity in their acoustic properties is consistent with the idea of a continuum of inter-speaker similarity, as suggested in Cavalcanti (2021). While identical twins may generally share a high degree of similarity in their voice/speech acoustic outcomes, the magnitude of intra-twin pair similarity can differ depending on the specific twin dyad being compared. This implies that the level of similarity between twin speakers is not necessarily uniform but can vary based on individual differences within twin pairs.

### 4.1 Threats to validity and future work

This study acknowledges the limitations inherent in its focus solely on male twins. We recognize that this choice may have implications for the findings and potentially limits the scope and applicability of our results. In future research, incorporating a more diverse participant pool, including female twins, could significantly enhance the breadth and applicability of the findings.

Additionally, while this study did not delve into the role of dialects in ASR performance, we understand the importance of such a factor. Expanding future research to include participants speaking different dialects could offer valuable insights, contributing to a more holistic understanding of the nuances in ASR technology.

Another avenue for further research could be to explore the effects of including twin data in the training of ASR models. However, our study shows that excluding twin voices results in improved ASR performance; it is possible that incorporating twin data in the training procedure could lead to better performance for speaker recognition for other twin pairs and the general population.

Finally, future studies could build on the present study's findings by exploring the impact of including twin voices on other speech tasks, such as reading or retelling. It would be interesting to investigate whether the inclusion or exclusion of twin data has a greater impact on ASR performance for certain speaking styles, where the degree of variation in speech patterns may differ.

## 5 Conclusions

In summary, the present study has shown that the inclusion of twin speakers in the ASR task can significantly challenge the performance of the system, with EER values ranging from 10 to 20% in the case of twin speakers, compared to values below 1% for testing conditions where no twin speakers were involved. The findings also suggest that specific twin pairs exhibit a greater degree of similarity than others, possibly due to variables that are beyond the scope of this research. This variability in the level of similarity between twin speakers could imply that some individuals pose a greater challenge for ASR systems.

The impact of speech sample duration on ASR performance has also been verified. It was observed that longer speech samples tended to produce lower EER and Cllr values, underscoring the relevance of sample duration in achieving more precise outcomes. Additionally, the results indicated a noticeable trend toward lower standard deviation values for both intra and inter-speaker similarity scores for larger samples compared to shorter ones. This trend was supported by the comparison between the 5 and 30 s sample sizes, suggesting that there is less variability in estimating speaker similarity/dissimilarity levels in longer stretches of speech than in shorter ones.

Furthermore, as the sample size increased, twin pairs demonstrated a marginal uptick in similarity, as indicated by a numerical rise in similarity scores from 5 to 30 s samples. Nevertheless, a parallel trend was observed in same-speaker comparisons: with a growing sample size, participants exhibited an increase in intra-speaker similarity. This result could clarify why, even though they exhibited a slight increase in similarity with larger sample sizes, the system achieved better separation between them.

Considering the significant drop in performance for different-speakers (DS) analyses restricted to twin pairs, in a hypothetical forensic application where there is a dispute between two identical twin brothers regarding the authorship of the questioned speech, it is recommended to use a voice database involving identical twin siblings, as applied in this research, in order to avoid obtaining artificially inflated likelihood ratios and high false positive rate.

## Data availability statement

The raw data supporting the conclusions of this article will be made available by the authors, without undue reservation.

## Ethics statement

The studies involving humans were approved by the Ethical Committee at Campinas State University (UNICAMP). The studies were conducted in accordance with the local legislation and institutional requirements. The participants provided their written informed consent to participate in this study.

## Author contributions

JC: Conceptualization, Data curation, Investigation, Writing—original draft, Writing—review & editing. RdS: Conceptualization, Data curation, Formal analysis, Investigation, Methodology, Software, Visualization, Writing—original draft, Writing—review & editing. AE: Funding acquisition, Project administration, Supervision, Writing—review & editing. PB: Conceptualization, Funding acquisition, Project administration, Resources, Supervision, Writing—review & editing.
